# Supplementary material for: Spatiotemporal characteristics and influencing factor analysis of universities’ technology transfer level in China: The perspective of innovation ecosystems
Source: PLoS One. 2022 Jun 30;17(6):e0270514. doi: 10.1371/journal.pone.0270514 (PMC9246116; doi:10.1371/journal.pone.0270514)
Supplement: Attachment — Submitted filename: Response to Reviewers.docx [file pone.0270514.s003.docx]

Response to Reviewer Comments

We would like to express my appreciation to you for suggesting how to improve our paper. We hope that the revised manuscript is now suitable for publication.

For reviewers’ comments, we have done following corresponding explanations and revisions.

**Reviewer 1**

**Point 1.** Please be clear about the research gap you are addressing. What is the aim of this paper and what is the missing in the literature and what is you are trying to clarify. Unfortunately, this is not easy to find out from text. Why don’t you be clear about the research gap and your research question?

**Response 1:** Thank you for your advice. We are very sorry that the research purpose of this paper is not clear in section 1. We have reorganized the content of introduction, enriched the literature review on UTT influencing factors, introduced the research gap of current research, and further clarified the research question and aim. (p. 4-5, line 66-108)

**Point 2.** Your literature review needs to be more focus in line with your research question. For instance, innovation ecosystem is not main topic of the paper but you have a section for it? Why, can’t you just summarize it in a paragraph.

**Response 2:** Thank you for your advice. We are sorry that the literature review is not in line with the research question. Under your advice, we have readjusted the structure of the article. On the one hand, we have reorganized the structure of introduction, moved the literature review to the section and added references on UTT level influencing factors, to make the literature review more focus in line with the research question. (p. 4-5, line 66-108) On the other hand, we have compressed the literature review of innovation ecosystem into a paragraph and moved it to the section of method together with the influencing factors of UTT (the initial manuscript 2.3). (p. 11-13, line 213-272)

**Point 3.** Please develop hypothesises (if you can) and link them to literature and your research model. Pls also draw a research model that can be useful to flow of your research logic.

**Response 3:** Thank you for your advice. We have added methodology model shown as Figure 1 to flow of the research logic. (p. 6, line 114-120) We are very sorry for it is difficult to put forward appropriate hypotheses because of the role of influencing factors different in neighboring provinces and few relevant research conclusions.

**Point 4.** Your methodology section is not very easy to follow and to understand. First, you used different models but no explanation why you have used them. Please elaborate on that. Also, create a smooth link between each section of methodology section.

**Response 4:** Thank you for your advice. We have added methodology model shown as Figure 1 and a paragraph to introduce the method to flow of the research logic. (p. 6, line 114-120) Before the introduction of UTT evaluation index method, a summary of evaluation methods has been added to illustrate the necessity of selecting comprehensive evaluation method. The steps of index screening and weighting method based on information contribution rate have been added to make the methodology part easier to understand. (p. 6-8, line 122-152) In addition, we have moved the influencing factors of UTT (the Initial manuscript 2.3) before the spatial econometric model of influencing factors, making them integrated and easy to understand. (p. 11-13, line 213-272)

**Point 5.** Please also explain why China has been chosen for this research. What makes China interesting for this study.

**Response 5:** Thank you for your advice. In section 1, we have added the reasons for choosing China for research. Over the past three decades China has made unprecedentedly large investments in university research and Chinese universities are now becoming major contributors to the global scientific community. However, it can be seen from the data that the proportion of UTT is still too low not to match its level of economic development. As the second largest university research system in the world, a better understanding of the complex ways of Chinese UTT is critical for scholars and policy makers globally. In the same way, improving Chinese UTT level can enhance innovation vitality and further promote economic development. Therefore, studies on UTT influencing factors are of great significance. (p. 3-4, line 57-65) China has a vast territory, and the economic development levels of various provinces are quite different. Due to the spatial heterogeneity of the impact of various factors on UTT, the current analysis of the influencing factors of UTT in China is insufficient. There is no relevant literature on spatiotemporal analysis of UTT level in China. (p. 5, line 101-14)

**Point 6.** Please try to update literature more with recent work. You can benefit from following new publications;

https://www.sciencedirect.com/science/article/abs/pii/S0160791X21001408

https://ieeexplore.ieee.org/abstract/document/9418622

https://www.sciencedirect.com/science/article/pii/S0160791X21002578

**Response 6:** Thank you for your advice. We have carefully studied the literature you listed, benefited a lot from it and quoted it in this article. These literatures also have important guiding significance for future research. (references [19,20,26])

**Reviewer 2**

**Point 1.** The paper would benefit from review by a native English speaker to assist with sentence construction and spelling. Examples of sentences not correct/clear:

- Contrary to expectation, the rate of commercialization 42 of university inventions to be too Low (line 42)

- “but it cannot fully reflect mutual influence of various regions based economic” (p.12. line 8)

**Response 1:** Thank you for your advice. We are very sorry for the errors in sentence and spelling making for not pleasant reading. We have revised the two problems you pointed out and asked a native English speaker to proofread.

The sentence “Contrary to expectation, the rate of commercialization 42 of university inventions to be too Low (line 42)” has been modified to “Over the past 20 years, universities have made steady progress in their efforts to facilitate the technology transfer process by working with industry [7], but UTT level is still remarkably low.” (p.3, line 44-45)

The sentence “but it cannot fully reflect mutual influence of various regions based economic” (p.12. line 8) has been modified to “but it does not sufficiently reflect the interaction of economic bases across regions”. (p.10, line 194-195)

**Point 2.** Among the regressors, the author(s) should include one (or more) variable(s) observing the specialization of the provinces’ economies

**Response 2:** Thank you for your advice. We are very sorry for not clarifying the point. Among the regressors, we used 3 indexes including GDP, high-tech industry development and financial development to observe the specialization of the provinces’ economies. (p. 12-13,line 248-271)

**Point 3.** author(s) should provide more technical details about the evaluation index screening and weighting used to build the UTT measure.

**Response 3:** Thank you for your advice. We are very sorry for not clarifying the technical details. We have added the steps of index screening and weighting method based on information contribution rate. Considering the repeatability factor of the paper, detailed calculation process can be found in the related reference [41]. (p. 7-8, line 140-150)

**Point 4.** Is the variable “Actual income in the year of UTT/Thousand yuan” (tab. 1) measured at constant prices? It should be. Otherwise, the UTT index might display a growing trend because of inflation.

**Response 4:** Thank you for your advice. We are very sorry for not clarifying the point. “Actual income in the year of UTT/Thousand yuan” measured at constant prices and the original data has been processed, which has been explained in the paper. (p. 8, line 150-152)

**Point 5.** Please provide references for using the spearman rank correlation coefficient as a tool to examine UTT trends

**Response 5:** Thank you for your advice. We are very sorry for the omission of references and have added references for using the spearman rank correlation coefficient as a tool to examine UTT trends. (p. 8, line 161)

**Point 6.** please generate figures’ captions and make figures more clear by introducing clear labels for the x and y axes.

**Response 6:** Thank you for your advice. We are very sorry for the missing graphics label. We redrew figures 3 and 4, and added ordinate labels to make the figures clearer.

**Point 7.** Please clarify that the analysis only allows the observation of robust ceteris paribus correlations among the variables included in the analysis.

**Response 7:** Thank you for your advice. We are very sorry for not clarifying the point. The problem of achievement transformation involved in this paper is a kind of exploratory problem, not a traditional economic forecasting problem. The fixed effect model is recommended for the estimation results, and its regression can obtain consistent results with strong robustness. Due to space limitation, we have modified the expression of robustness in this paper and will continue to study the transformation conditions to verify robustness in the future. (p. 20, line 375-376)

**Point 8.** The author(s) focus their comments on statistical significance results. Instead, it would be essential to provide detailed comments about the magnitude of the coefficients calculated through the regression analysis. Commenting on the coefficients will provide essential elements about the economic significance of the findings.

**Response 8:** Thank you for your advice. We are very sorry for not clarifying the point. Since the paper did not adopt normalization treatment for various index data, we did not compare the regression coefficients of various influencing factors. We will try to conduct comparative analysis on the regression coefficients of each variable in the future research.

**Point 9.** The interpretation of the results concerning the financial development variable requires more argument (p.22).

**Response 9:** Thank you for your advice. We are very sorry for not clarifying the point. This paper adds the literature on explaining the results of financial development variables. The direct impact of financial development on UTT is negative and significant at the level of 5%, which may be related to the fact that financial development in recent years has focused on virtual economies such as virtual real estate, insufficient support for the real economy where UTT is located. In addition, the current financial system is not robust and the financial scale is opposite to financial support, the expansion of financial scale reduces the financial support effect [66-68]. (p. 22-23, line 398-403)

**Point 10.** The interpretation of the results concerning “Technology intermediary” is unclear to me. To the best of my understanding, the variable is not statistically significant, while the comment assumes it is significant.

**Response 10:** Thank you for your advice. We are very sorry for not clarifying the point. This paper explains the difference between the result and hypothesis of " Technology Intermediary ": because the current technology market development is still in its infancy [69], the support to UTT is still limited. (p.23, line 404-408)

**Point 11.** “The possible reason is that the influence of these factors on UTT has an inverted U-shaped threshold effect”. (p. 24, line 4). Please provide a more precise illustration of this interpretation. To the best of my understanding, the findings demonstrate that some of the factors' effects vary according to the dependent variable's level.

**Response 11:** Thank you for your advice. We are very sorry for not clarifying the point. The inverted U-shaped conclusion in the paper is the author's estimate, without rigorous data demonstration. In consideration of the rigor of the article, we deleted the description of inverted U shape and carried out further research after consideration.

**Minor comments**

- please provide details about how the effective patent application rate (line 49 is calculated

**Response 12:** Thank you for your comments. We are very sorry for the wrong word. The effective patent application rate in line 49 should be corrected to the effective patent application rate. The data comes from “China Intellectual Property Survey Report 2019” by China National Intellectual Property Administration. (p. 3, line 52-53)

- The analysis allows inspecting the correlates of UTT in the Chinese provinces while it cannot provide any helpful information about “the driving mechanism of influencing factors” (line 58)

**Response 13:** Thank you for your comments. We are very sorry for the inaccurate expression. We changed “the driving mechanism of influencing factors” to “the impact of various factors on UTT”. (p.6, line 108)

- the “reference source not found” errors appears at p. 4

**Response 14:** Thank you for your comments. We are very sorry for the error at p.4. We rearranged and checked the reference labels of the references.

- “most of them” (p. 6 line 9); most of who?

**Response 15:** Thank you for your comments. We are very sorry for not clarifying the point. We have asked a native English speaker to help us to assist with sentence construction and spelling.

The phrase “and most of them” has been modified to “most of which”, referring to the evaluation of UTT capability. (p.6, line 122-123)
