# Supplementary material for: Spatiotemporal characteristics and influencing factor analysis of universities’ technology transfer level in China: The perspective of innovation ecosystems
Source: PLoS One. 2022 Jun 30;17(6):e0270514. doi: 10.1371/journal.pone.0270514 (PMC9246116; doi:10.1371/journal.pone.0270514)
Supplement: Attachment — Submitted filename: Response to Reviewers.docx [file pone.0270514.s004.docx]

Response to Comments

We would like to express my appreciation to you for suggesting how to improve our paper. We hope that the revised manuscript is now suitable for publication.

For additional editor and reviewers’ comments, we have done following corresponding explanations and revisions.

Additional Editor Comments:

**Comment 1:** I am still struggling to understand what is the research gap in the literature. According to what you wrote, “there is no relevant literature on spatiotemporal analysis of UTT level in China”. However, this is based on a review of the literature that is missing some relevant recent studies:
• Yuan et al. (2018). Dynamic capabilities, subnational environment, and university technology transfer
• Wu et al. (2021). Does social trust stimulate university technology transfer? Evidence from China
• Li and Tang (2021). A dynamic capabilities perspective on pro-market reforms and university technology transfer in a transition economy

**Response 1:** Thank you for pointing out the shortcomings. We have carefully studied the literature you recommended and conducted some literature searches to put forward the research gaps and innovations.

The following discussion have been added in the revised manuscript here for your easy reference: *Some scholars have noted the heterogeneous impact of subnational environment [15], social trust [16], and pro-market reforms [17] on UTT. Hou et al. discover that the moderating role of intermediary organizations in academia-industry cooperation and industrial innovation varies in inland and coastal areas [18]. The sustainable development and transformation of knowledge and innovation should be considered as a dynamic and systematic coevolutionary process [19]. Considering spatial relationship in regression analysis becomes a popular paradigm. The heterogeneous impact of regional innovation ecosystems on UTT need to be recognized deeper with spatial analysis as well. The innovations and contributions of this paper are: (1) establishing a comprehensive evaluation index system of UTT level based on the evaluation index screening and weighting method based on information contribution rate; (2) analyzing the heterogeneous impact of innovation actors on UTT from the perspective of innovation ecosystem.* (p.4, line 67-77)

**Comment 2:** Point 2 from Reviewer#1 did not suggest deleting entirely your literature review section, but only to reduce section 2.1. You should keep section 2 as your literature review section, and strengthening it with more studies on UTT in China – in order to finetune and narrow your work, and create a better linkage between research gap, research question, literature, and methodological approach
As a result of the removal of the whole literature review section, actually section 2.3 includes a mix of method and literature review. This is rather confusing.

**Response 2:** Thank you for your guidance. In the last revision, we referred to some literature structures and decomposed the literature review part into the introduction and methods parts. As you point out, we have recognized that such a structure is not common, and restored the literature review section. At the same time, we have added some literatures in the literature review and proposed hypotheses based on the literature review. (p.4-8, line 82-167)

**Comment 3:** You have not addressed one key issue raised by Reviewer#1:
• Point 3: your reply “it is difficult to put forward appropriate hypotheses because of the role of influencing factors different in neighboring provinces and few relevant research conclusions” is not satisfactory: hypotheses are based on the literature, not on the presence different influencing factors in different provinces. Moreover, why this should be a problem in the development of hypotheses?
**Response Point 3:** Thanks for your guidance. We have seriously reflected on your suggestions, put forward hypotheses in the literature review section, and explained whether the hypotheses are valid in the result analysis.

The following hypotheses have been added in the revised manuscript here for your easy reference:

*H1: Provincial UTT Level shows spatial autocorrelation in China; H2: The factors in innovation ecosystem have positive impact on UTT; H3: The influence of the factors on UTT level is spatially heterogeneous in China.* (p.6, line 113, p.8, line 116-117, p17, line301-304, and p21, line350-351)

**Comment 4:** You did not address some of the key issues raised by Reviewer#2:
• Point 2: The specialization of the provinces’ economies should be based on a survey on the local enterprises based on their NACE sectors

**Response** **Point 2:** Thank you for your advice. According to your suggestion, we have searched literatures related to university technology transfer and economic specialization, only a small amount of literature addressed both keywords, such as University technology transfer and the evolution of regional specialization_ the case of Turin，Co-evolution patterns of university patenting and technological specialization in European regions. These studies build a framework of the co-evolution of university technology transfer and regional specialization, and we are deeply inspired and provide good ideas for further in-depth research of the interaction between innovation ecosystem and university technology transfer. Moreover, these studies also prove the necessity of heterogeneity analysis of the influencing factors of university technology transfer. For example, University technology transfer and the evolution of regional specialization the case of Turin states: The geographical closeness between universities and firms is important because the exchanged knowledge is cumulative, localized, and tacit in nature allowing local firms to access the results of academic research more easily. Geographical proximity may also strengthen other forms of proximity such as cognitive, organizational, and technological closeness. Other literatures have also reached some similar conclusions, such as the absorptive capacity of firms may affect the effective transfer of knowledge from universities to local firms, the transfer of complex knowledge is affected by the technological proximity of economic agents within the local system. However, after careful discussion and research, we believe that economic specialization (or regional specialization) is not the main body of the innovation ecosystem in the framework of this paper, nor is it an essential influencing factor. Therefore, the paper does not regard economic specialization as research variable for the time being.

In addition, we refer to the relevant variable classifications of classified statistical agencies such as the National Bureau of Statistics of China and the State Administration for Market Regulation (refer to the website: <http://www.stats.gov.cn/tjsj/ndsj/2019/indexeh.htm>)

• Your reply to Point 8 is not satisfactory: you do not have to compare the coefficients of the variables, you need to comment on the meaning (in economic and statistical terms) of the single coefficients

**Response Point 8:** Thank you for your advice. We are very sorry for not clarifying the coefficients meaning. According to your suggestion, we have supplemented the analysis of the meaning of the single coefficients of the estimated influencing factors in Table 8.

The following discussion have been added in the revised manuscript here for your easy reference: *The UTT of the province decreases by 0.0335 units along with the government investment increases by 1 unit, which is not significant. While, the UTT of neighboring provinces decreases by 0.969 at a significant level of 1%*; *The direct impact of financial development on UTT is negative and significant at the level of 5%. The UTT of the province decreases by 0.0177 units in the wake of the financial development increases by 1 unit, while the UTT of neighboring provinces increases by 0.0471, which is not significant*. (p.21-24, line 353-355, line 365-366,line 368, line 374-376, line 399-401, line 404-407)

• Point 9: The interpretation of the results concerning the financial development variable is still not sufficient

**Response Point 9:** Thank you for your advice. We are very sorry for not clarifying the interpretation of the results concerning the financial development variable. To better explain the mechanism of action of financial development, we have added three literatures illustrating the opposite of financial size and financial support.

The following explanations have been added in the revised manuscript here for your easy reference: *This seems to be kind of “Vanishing Effect” [68], financial deepening or private sector credit exceeds a certain size when the financial contribution to economic growth disappears. Financial development could have a negative impact on economic growth since private credit exceeds 110% of GDP[69]. The role of financial development in economic growth has been found as an inverted U-shaped relationship[70].* (p.22, [68-70],line 379-383)

• Your response to Point 10 is unclear and does not address the comment from the reviewer
**Response Point 10:** Thank you for your advice. We are very sorry for not clarifying the point. In table 8, *Int* variable (Technology Intermediary) has positive effects on UTT of province and negative effects on neighboring provinces, but does not pass the significance test, indicating that technology intermediary fails to have a significant positive impact on province nor significant negative impact on neighboring provinces. On the one hand, the value creation capability of technology intermediaries is clearly. Mature technology transfer channels, sound achievement evaluation mechanism and professional technical manager team can effectively promote UTT. Technology intermediaries are the link between universities and technology-demanding enterprises, creating value with their own professional services in the process of technology transfer. Technology intermediaries can facilitate the transformation of technology, promote the integration of industry-academia-research and advance the process of technology marketization, thus greatly improving the efficiency of UTT. On the other hand, various factors constrain the role played by technology intermediaries in the UTT process. The current technology market development is still in its infancy [74] and the improvement of technology intermediary capacity requires coordinated cooperation between government and other organization, the support to UTT is still limited, the impact of technology intermediary on the UTT has yet to be further strengthened. (p.22-23, line 385-397)

Minor points:
• Line 36: the acronym for UTT is described in the abstract, but not in the main text. The first time you use it, you need to clarify what the acronym stands for

**Response 1:** Thank you for your advice. We are very sorry for this. In introduction where we first use it, we have added specific meaning of the acronyms. (p.2, line 32)

• Line 37 and 46: I do not understand why citing twice the paper of Trippl et al. (2015) in parenthesis
• The first section in the Introduction is based on several citations from the paper of Trippl et al. (2015): this is rather unusual

**Response 2:** Thank you for your advice. We are very sorry for not clarifying the point. In the revise revision “Revised Manuscript with Track Changes”, due to some technical factors (using word's revision mode), there were errors in the marking of references, most of them were marked as [1], but these are different literatures, and the new revised version will not have this problem again.

• Line 254: what does it mean that the UTT evaluation method is based on “the basis of the full-text analysis”?

**Response 3:** Thank you for your advice. We are very sorry for not clarifying the point. This expression is kind of chronic statement with no specific meaning, so we have deleted this sentence. Sorry for confusing you.

• When presenting Table 2, you should include a column with a short description of the variables and their unit of measure

**Response 4:** Thank you for your advice. We are very sorry for not clarifying the point. According to your suggestion, we have added Table 2. (p.14, line 273)

The following is Table 2 have been added in the revised manuscript here for your easy reference:

***Table 2. Proxy Variables for Influencing Factors***

| ***Influence Factor*** | ***Proxy Variable*** |
| --- | --- |
| *UTT Level (UTT)* | *Results by the evaluation index screening and weighting method based on information contribution rate* |
| *Government (Gov)* | *R&D investment intensity* |
| *Industry (Ind)* | *The operating income of high-tech industry per 10,000 people (10, 000 yuan)* |
| *Financial Institute (Fin)* | *The ratio of balance of deposits to loans of financial institutions at the end of the year to GDP* |
| *Technology Intermediary (Int)* | *technology market turnover per capita (10, 000 yuan)* |
| *Economic (Eco)* | *per capita GDP (10, 000 yuan)* |
| *Informatization (Inf)* | *the number of Internet interfaces per 10,000 people* |
| *Openness (Open)* | *the investment amount of foreign-invested enterprises per 10,000 people (10, 000 dollars)* |

**Closing comment to Editor:** We hope the revised manuscript is now acceptable to you. If not, we are glad to receive any further feedback which we shall continue to apply our best effort to address.
