# Supplementary material for: Spatiotemporal characteristics and influencing factor analysis of universities’ technology transfer level in China: The perspective of innovation ecosystems
Source: PLoS One. 2022 Jun 30;17(6):e0270514. doi: 10.1371/journal.pone.0270514 (PMC9246116; doi:10.1371/journal.pone.0270514)
Supplement: Attachment — Submitted filename: Response to Reviewers.docx [file pone.0270514.s006.docx]

Response to Comments

We would like to express my appreciation to you for suggesting how to improve our paper. We hope that the revised manuscript is now suitable for publication.

For Academic Editor’ comments, thank you for your valuable and thoughtful comments. We have sought independent editorial help from a professional proofreader to improve writing style and correct language. For Journal Requirements, we checked the references again and attached the last revision and the results of this check.

Journal Requirements:

Thank you for your advice. According to your suggestion, we have checked reference list and refined the content and format according to the requirements of the journal. We have deleted Reference 54, because of repeat with Reference 52. The specific modifications are as follows.

**Reference 1:** Added magazine abbreviations and article Issue.

*Trippl M, Sinozic T, Smith HL. The role of universities in regional development: Conceptual models and policy institutions in the UK, Sweden and Austria. Eur Plan Stud. 2015; 23(9): 1722-1740.*

**Reference 3:** Added article specific time of publication.

*Han J. Technology Commercialization through Sustainable Knowledge Sharing from University-Industry Collaborations, with a Focus on Patent Propensity. Sustainability. 2017 Oct 8. doi: 10.3390/su9101808.*

**Reference 4:** Added magazine abbreviations.

*Etzkowitz H, Leydesdorff L. The dynamics of innovation: From national systems and “mode 2” to a triple helix of university–industry–government relations. Res Policy. 2000; 29(2):109-123.*

**Reference 5:** Added magazine abbreviations.

*Harman G． Australian university research commercialization: perceptions of technology transfer specialists and science and technology academics．J High Educ Policy Manag. 2010;32(1):69-83．*

**Reference 6:** Added magazine abbreviations and article Issue.

*Buenstorf G． Is commercialization good or bad for science? Individual - level evidence from the Max Planck Society．Res Policy. 2009; 38(2):281-292．*

**Reference 7:** Changed article published year.

*Muscio A. What drives the university use of technology transfer offices? Evidence from Italy. J Technol Transf. 2009; 35(2): 181-202.*

**Reference 9:** Added magazine abbreviations.

*Markman GD, Phan PH, Balkin DB, Gianiodis PT. Entrepreneurship and university-based technology transfer. J Bus Ventur. 2005;20(2):241-263.*

**Reference 10:** Added magazine abbreviations.

*Sampat B N. Patenting and US academic research in the 20thcentury: The world before and after Bayh-Dole. Res Policy. 2006;35(6):772-789．*

**Reference 13:** Changed citation format and added article Issue.

*Chen, A., Patton, D. & Kenney, M. University technology transfer in China: a literature review and taxonomy. J Technol Transf. 2016; 41(5): 891-929.*

**Reference 14:** Added authors and magazine abbreviations.

*Yang W, Yu X, Wang D, Yang JR, Zhang B. Spatio-temporal evolution of technology flows in China: patent licensing networks 2000-2017. J Technol Transf. 2021,46(5):1674-1703.*

**Reference 15:** Added authors and magazine abbreviations.

*Yuan CH, Li Y, Vlas CO, Peng MW. Dynamic capabilities, subnational environment, and university technology transfer. Strateg Organ. 2018; 16(1):35-60.*

**Reference 16:** Added magazine abbreviations and article doi.

*Wu Y, Huang W, Deng L. Does social trust stimulate university technology transfer? Evidence from China. PLoS One. 2021; 16(8). doi: 10.1371/journal.pone.0256551.*

**Reference 17:** Added article doi.

*Li, Y; Tang, YJ. A dynamic capabilities perspective on pro-market reforms and university technology transfer in a transition economy. Technovation.2021;103. doi: 10.1016/j.technovation.2021.102224.*

**Reference 18:** Added authors and magazine abbreviations.

*Hou BJ, Hong J, Chen Q, Shi X, Zhou Y. Do academia-industry R&D collaborations necessarily facilitate industrial innovation in China? The role of technology transfer institutions. Eur J Innov Manag. 2019; 22(5):717-746.*

**Reference 20:** Added magazine abbreviations.

*Xiong X, Yang GL, Guan ZC. Assessing R&D efficiency using a two-stage dynamic DEA model: A case study of research institutes in the Chinese Academy of Sciences. J Informetr. 2018; 12(3):784-805.*

**Reference 21:** Added authors and magazine abbreviations.

*Gao Q, He F, Moosa A, Lv Q. The Efficiency of Technology Transfer in Chinese Key Universities. J Sci Ind Res. 2019; 78(9):582-588.*

**Reference 22:** Added magazine abbreviations.

*Son H, Chung Y, Yoon S. Is the alignment between public research organizations’ R&D competence and policies really critical for technology transfer? Sci Public Policy. 2021; 48(1):93-104.*

**Reference 23:** Added magazine abbreviations.

*Di F. Transfer Benefit Evaluation on University S&T Achievements Based on Bootstrap-DEA. Educ Sci-Theory Pract. 2018; 18(5):1125-1137.*

**Reference 26:** Added magazine abbreviations.

*Lampe HW, Hilgers D. Trajectories of efficiency measurement: A bibliometric analysis of DEA and SFA. Eur J Oper Res. 2015; 240(1):1-21.*

**Reference 27:** Added article specific time of publication.

*Li F, Zhang S, Jin YH. Sustainability of University Technology Transfer: Mediating Effect of Inventor's Technology Service. Sustainability. 2018 Sep 13. doi:10.3390/su10062085.*

**Reference 29:** Added authors and magazine abbreviations.

*29.	Calcagnini G, Favaretto I, Giombini G, Perugini F, Rombaldoni R. The role of universities in the location of innovative start-ups. J Technol Transf. 2016;41(4):670-693.*

**Reference 30:** Added authors and magazine abbreviations.

*30.	Colombelli A, De Marco A, Paolucci E, Ricci R, Scellato G. University technology transfer and the evolution of regional specialization: the case of Turin. J Technol Transf. 2021; 46(4):933-960.*

**Reference 31:** Added magazine abbreviations.

*Boschma, R. Proximity and innovation: A critical assessment. Reg Stud. 2005; 39(1):61-74.*

**Reference 32:** Added magazine abbreviations.

*Siegel DS, Waldman DA, Atwater LE, Link AN. Toward a model of the effective transfer of scientific knowledge from academicians to practitioners: qualitative evidence from the commercialization of university technologies. J Eng Technol Manage. 2004; 21(1-2):115-142.*

**Reference 33:** Added magazine abbreviations.

*Gregorio DD, Shane S. Why do some universities generate more start-ups than others? Res Policy. 2003;32(2):209-227.*

**Reference 35:** Added magazine abbreviations.

*Caldera A, Debande O. Performance of Spanish universities in technology transfer: An empirical analysis. Res Policy. 2010; 39(9):1160-1173.*

**Reference 36:** Added magazine abbreviations, article specific time of publication and doi.

*Sun, Chia-Chi. Evaluating the Intertwined Relationships of the Drivers for University Technology Transfer. Appl Sci-Basel. 2021 Nov 25; 20(11). doi: 10.3390/app11209668.*

**Reference 37:** Added authors, magazine abbreviations and article specific time of publication.

*Ar IM, Temel S, Dabic M, Howells J, Mert A, Yesilay RB. The Role of Supporting Factors on Patenting Activities in Emerging Entrepreneurial Universities. IEEE Trans Eng Manage. 2021 Dec 28. doi:10.1109/TEM.2021.3069147.*

**Reference 38:** Added authors, magazine abbreviations and Volume.

*Jonek-Kowalska I, Musiol-Urbanczyk A, Podgorska M, Wolny M . Does motivation matter in evaluation of research institutions? Evidence from Polish public universities. Technol Soc. 2021; 67. doi: 10.1016/j.techsoc.2021.101782.*

**Reference 39:** Added magazine abbreviations and pages.

*Lafuente E, Berbegal-Mirabent J. Assessing the productivity of technology transfer offices: an analysis of the relevance of aspiration performance and portfolio complexity. J Technol Transf. 2019; 44(3): 778-801.*

**Reference 40:** Added magazine abbreviations and Volume.

*Wonglimpiyarat, Jarunee. The innovation incubator, university business incubator and technology transfer strategy: The case of Thailand. Technol Soc. 2016;46: 18-27.*

**Reference 41:** Added authors.

*Escobar ESO, Berbegal-Mirabent J, Alegre I, Velasco OGD. Researchers’ willingness to engage in knowledge and technology transfer activities: an exploration of the underlying motivations. R&D Management. 2017; 47(5):715-726.*

**Reference 42:** Added article Issue.

*Belenzon, S.; Schankerman, M. University Knowledge Transfer: Private Ownership, Incentives, and Local Development Objectives. J Law Econ. 2009; 52(1): 111-144.*

**Reference 44:** Added authors, magazine abbreviations and article specific time of publication.

*Vlaisavljevic V, Medina CC, Looy BV. The role of policies and the contribution of cluster agency in the development of biotech open innovation ecosystem. Technol Forecast Soc Chang. 2020 May 14. doi: 10.1016/j.techfore.2020.119987.*

**Reference 47:** Added article specific time of publication.

*Zhao JY, Wu GD. Evolution of the Chinese Industry-University-Research Collaborative Innovation System. Complexity, 2017 May 17. doi:10.1155/2017/4215805.*

**Reference 51:** Added magazine abbreviations.

*Munari F, Sobrero M, Toschi L. The university as a venture capitalist? Gap funding instruments for technology transfer. Technol Forecast Soc Chang. 2018; 127:70-84.*

**Reference 52:** Added magazine abbreviations.

*Villani E, Rasmussen E, Grimaldi R. How intermediary organizations facilitate university-industry technology transfer: A proximity approach. Technol Forecast Soc Chang. 2017;114:86-102.*

**Reference 53:** Added magazine abbreviations.

*Al-Tabbaa O, Ankrah S. Social capital to facilitate 'engineered' university-industry collaboration for technology transfer: A dynamic perspective. Technol Forecast Soc Chang. 2016;104:1-15.*

**Reference 54:** Added authors, magazine abbreviations and article specific time of publication.

*Temel S, Dabic M, Ar IM, Howells J, Mert A, Yesilay RB. Exploring the relationship between university innovation intermediaries and patenting performance. Technol Soc. 2021 Aug 13. doi: 10.1016/j.techsoc.2021.101665.*

**Reference 56:** Added article specific time of publication.

*Fang HN, Yang Q, Wang JM, et al. Coupling Coordination between Technology Transfer in Universities and High-Tech Industries Development in China. Complexity. 2021 Aug 1. Doi:10.1155/2021/1809005.*

**Reference 57:** Added magazine abbreviations.

*Dikbas F. A New Two-Dimensional Rank Correlation Coefficient. Water Resour Manag. 2018; 32(5):1539-1553.*

**Reference 58:** Added magazine abbreviations and article specific time of publication.

*Stephanou M, Varughese M. Sequential estimation of Spearman rank correlation using Hermite series estimators. J Multivar Anal. 2021 Oct 10, 186, doi: 10.1016/j.jmva.2021.104783.*

**Reference 59:** Added magazine abbreviations and article specific time of publication.

*Yuan YY; Han ZL. Structural characteristics and proximity comparison of China's urban innovation cooperation network. PLoS One. 2021 Aug 26. doi: 10.1371/journal.pone.0255443.*

**Reference 60:** Added article specific time of publication.

*Tang, S, Zhang JX, Niu FQ. Spatial-Temporal Evolution Characteristics and Countermeasures of Urban Innovation Space Distribution: An Empirical Study Based on Data of Nanjing High-Tech Enterprises. Complexity. 2020 Nov 4. doi: 10.1155/2020/2905482.*

**Reference 61:** Added magazine abbreviations.

*Broekel T, Boschma R. Knowledge networks in the Dutch aviation industry: the proximity paradox. J Econ Geogr. 2012;12(2): 409–433.*

**Reference 62:** Added magazine abbreviations.

*Xiong JB, Liu YQ, Lin XG. Geographic distance and pH drive bacterial distribution in alkaline lake sediments across Tibetan Plateau. Environ Microbiol. 2012;14(9):2457-2466.*

**Reference 63:** Added article specific time of publication.

*Zheng DF, Hao S, Sun CZ. Spatial Correlation and Convergence Analysis of Eco-Efficiency in China. Sustainability. 2019 Jul 9. doi:10.3390/su11092490.*

**Reference 64:** Added magazine abbreviations.

*Arbia G. Spatial Econometrics: Statistical Foundations and Applications to Regional Convergence. J Reg Sci. 2007; 47(3):646-648.*

**Reference 65:** Added authors and article specific time of publication.

*Chen Y, Shi H, Ma J, Shi V. The Spatial Spillover Effect in Hi-Tech Industries: Empirical Evidence from China. Sustainability. 2020 Apr 14. doi:10.3390/su12041551.*

**Reference 66:** Added magazine abbreviations.

*Hsu PH, Tian X, Xu Y. Financial development and innovation: Cross-country evidence. J financ econ. 2014; 112(1):116-135.*

**Reference 67:** Added magazine abbreviations.

*Rousseau, P. L., Wachtel, P. What Is Happening to the Impact of Financial Deepening on Economic Growth. Econ Inq. 2011; 49(1):276-288.*

**Reference 68:** This reference have been removed and replace it with relevant current references as follows:

*Mensi W, Hammoudeh S, Tiwari AK, Al-Yahyaee KH. Impact of Islamic banking development and major macroeconomic variables on economic growth for Islamic countries: Evidence from panel smooth transition models. Econ Syst. 2020 May 8. doi: 10.1016/j.ecosys.2019.100739.*

**Reference 70:** Added authors, magazine abbreviations and article specific time of publication.

*Gao X, Ji Z, Ahmad F, Draz MU. Financial Support and Growth of Township and Village Enterprises in China: Fresh Evidence From Regional Analyses. Sage Open. 2019 Jul 9, 9(2). doi:10.1177/2158244019858444.*

**Reference 71:** Added magazine abbreviations.

*Zhang H, Ding D, Ke L. The Effect of R&D Input and Financial Agglomeration on the Growth Private Enterprises: Evidence from Chinese Manufacturing Industry. Emerg Mark Financ Trade. 2019; 55(10):2298-2313.*

**Reference 72:** Added magazine abbreviations.

*Karlsson H K, Mnsson K, Hacker S. Revisiting the nexus of the financial development and economic development: new international evidence using a wavelet approach. Empir Econ. 2021; 60(5): 2323-2350.*

**Reference 73:** Added magazine abbreviations.

*Demirel P, Kesidou E. Sustainability-oriented capabilities for eco-innovation: Meeting the regulatory, technology, and market demands. Bus Strateg Environ. 2019; 28(5):847-857.*
